# Associations of clinical and circulating metabolic biomarkers with low physical fitness and function in adults with chronic lymphocytic leukemia

**DOI:** 10.3389/fonc.2022.933619

**Published:** 2022-08-03

**Authors:** Andrea Sitlinger, Michael A. Deal, Erwin Garcia, Margery Connelly, Dana Thompson, Tiffany Stewart, Grace Macdonald, Erik D. Hanson, Megan Neely, Ben Neely, Ashley Artese, J. Brice Weinberg, Danielle Brander, David B. Bartlett

**Affiliations:** ^1^ Hematologic Malignancies and Cellular Therapies, Duke University Medical Center, Durham, NC, United States; ^2^ Division of Medical Oncology, Duke University Medical Center, Durham, NC, United States; ^3^ Laboratory Corporation of America Holdings (Labcorp), Morrisville, NC, United States; ^4^ Division of Hematology, Duke University Medical Center and VA Medical Center, Durham, NC, United States; ^5^ Department of Exercise and Sport Science, University of North Carolina, Chapel Hill, NC, United States; ^6^ Department of Biostatistics and Bioinformatics, Duke University Medical Center, Durham, NC, United States; ^7^ Duke University Aging Center, Duke University Medical Center, Durham, NC, United States; ^8^ School of Bioscience and Medicine, University of Surrey, Guildford, United Kingdom

**Keywords:** chronic lymphocytic leukemia (CLL), lipids and lipoproteins, metabolism, physical fitness, physical function

## Abstract

Many patients with chronic lymphocytic leukemia (CLL) experience physical dysfunction and low overall fitness. It remains unknown what factors drive CLL physical dysfunction. We assessed physical function and metabolic lipoprotein panels in 106 patients with CLL. In univariate analyses of clinical factors, a longer time since diagnosis was associated with a higher likelihood of dysfunctional aerobic fitness (OR = 3.56, 95% CI: 1.37–9.22; *p* = 0.002) and physical performance (SPPB: OR = 2.03, 95% CI: 1.20–3.44; *p* = 0.004). Having received treatment was associated with a higher likelihood of dysfunctional aerobic fitness (OR = 1.57, 95% CI: 1.02–2.40; *p* = 0.036), SPPB (OR = 1.85, 95% CI: 1.13–3.03; *p* = 0.011) and grip strength (OR = 1.67, 95% CI: 1.10–2.55; *p* = 0.015). We found that several small HDL particle parameters, higher levels of citrate (OR = 2.01, 95% CI: 1.22–3.31; *p* = 0.030), and lower levels of hemoglobin (OR = 0.50, 95% CI: 0.31–0.82; *p* = 0.030) were associated with a higher likelihood of dysfunctional aerobic fitness. Multivariable least absolute shrinkage and selection operator (LASSO)-penalized regression analyses using variable importance measures (VIM) showed that 7.8-nm HDL particles (VIM = 1.000) and total HDL particle levels (VIM = 1.000) were more informative than clinical measures for the odds of dysfunctional aerobic fitness and 6-min walk functional fitness, respectively, while 10.3-nm HDL particles (VIM = 0.383) were more informative for grip strength. Time since diagnosis (VIM = 0.680) and having received treatment (VIM = 0.490) were more informative than lipoprotein measures for the odds of having dysfunctional SPPB. Taken together, we establish significant relationships between clinical and metabolic factors and physical characteristics that might prompt early use of ancillary support services.

## Introduction

Chronic lymphocytic leukemia (CLL), the most common adult leukemia, is incurable. With a median diagnosis age of 71 years, there are more than 20,000 new cases/year, 4,000 deaths/year, and over 130,000 adults living with the disease in the USA (1–3). CLL has a highly variable clinical course, with overall survival at 5 years ranging from 23% to 93% (4). CLL patients have a 20%–30% increased risk of secondary malignancies and autoimmune diseases, and bacterial and viral infections are the leading causes of hospitalization and death in CLL patients (5–8). Most CLL patients are diagnosed with a low-grade leukemia and have a period of treatment-free observation before needing treatment (9, 10). Current treatment options, including chemoimmunotherapy and targeted therapies, have improved survival, but immunosuppression persists, and cancer-related comorbidities are common. Overall, CLL patients have a shorter life expectancy than age-matched healthy subjects (4, 11).

As such, adults with CLL have poor resilience to cancer and age-associated stressors (12, 13). Further complicating resilience, approximately 60%–70% of older adults with CLL may be pre-frail or frail, compared to approximately 11% in community-dwelling older adults (14, 15). A dominant characterization of frailty is low physical and functional fitness (12). For those with CLL, low overall physical fitness and physical dysfunction are predictive of poor survival following the commencement of cancer-specific treatment (16). Conversely, higher physical activity levels and general fitness for patients with blood cancer are associated with better quality of life and physical function (13, 17, 18) (albeit these studies included very few CLL participants). This suggests that CLL is likely a significant cause of physical fitness declines and that lifestyle interventions that target reductions may improve outcomes. However, it is unclear to what extent these improvements would occur in CLL patients, what the mechanisms are, and how the leukemia cells, the treatments, or standard demographics such as age and body composition contribute to physical declines. One potential mechanism driving reduced fitness in CLL may be how the metabolic profile of leukemia cells affects healthy tissues and organs, including skeletal and cardiac muscle functions. Specifically, CLL cells are lipid dependent, using fatty acids and cholesterol for their survival (19). Consequently, the balance between LDL, HDL, triglycerides, and other lipoprotein subclasses may be potentially depriving muscle and other tissues of fuel sources (20). Until we can better understand the causes of accelerated physical declines in CLL, optimal interventions that improve quality of life and clinical outcomes will remain limited. Therefore, we aimed (1) to assess the relationships between circulating metabolic biomarkers and clinical indices, and (2) to determine factors that increase the likelihood of low physical fitness in CLL.

## Methods

### Patient characteristics

We completed a clinical observational study in the CLL clinic of the Duke Cancer Center during patients’ regularly scheduled appointments. We assessed physical activity questionnaires and physical fitness and function in 144 CLL patients between July 2017 and March 2018. Of these, we analyzed 106 patients who provided adequate blood for analyses of biomarkers by nuclear magnetic resonance (NMR) spectroscopy. Patients were approached during their clinical visits and, if they agreed to take part, were informed of the study by the study team. This study was approved by the Duke University Medical Center Institutional Review Board in accordance with the Declaration of Helsinki, and all patients provided written informed consent before study testing.

### Clinical data

We obtained clinical indices from the patient’s medical records. We grouped patients into those who had never received treatment for their CLL (Treatment Naïve: *N* = 47) and those who had received CLL therapy (Treated: *N* = 59). Clinical indices included the CLL-IPI Score, calculated as previously described (4), most recent RAI staging, disease duration, cytogenetics [i.e., fluorescent *in situ* hybridization (FISH) for CLL], *IGHV* mutation status, *TP53* mutation status, CD38 expression levels, lactate dehydrogenase levels, β2-microglobulin levels, and complete blood counts and leukocyte differential counts. Any patient who had 17p deletion or mutation, 13q deletion, or multiple cytogenetic abnormalities was considered to have unfavorable cytogenetics.

### Physical performance and fitness

We have described physical performance and fitness measures previously (21). Briefly, patients completed a short battery of standardized physical performance tests conducted by trained specialists. These included the 6-minute walk test (6MWT), the short physical performance battery (SPPB), and grip strength. After physical testing, patients completed the Stanford Brief Activity Survey (SBAS). The SBAS is a two-item questionnaire that asks participants to classify both their on-the-job and leisure-time activity. We classified each participant into one of five physical activity categories ranging from “inactive” to “very hard” intensity (22). We measured height and weight before testing and blood pressure and resting heart rate following 10 min of seated rest. Finally, we estimated the patient’s peak aerobic capacity [eVO_2peak_ (ml/kg/min)] using a validated equation that incorporates 6MWT distance, resting heart rate, weight, sex, and age (23).

### Classification of dysfunctional physical performance and fitness

We classified patients into low (i.e., dysfunctional) and normal (i.e., functional) physical fitness/function levels from classifications found in relation to frailty or increased risk of worse clinical outcomes; specifically, low aerobic fitness as ≤15 ml/kg/min (24), low 6MWT distance as ≤400 meters (m), low SPPB as a score ≤10 (25), and low SBAS as completing less than moderate intensity physical activity levels (22). For low grip strength, we stratified patients according to sex and BMI (26). For male patients, we classified low if the following was true (1) BMI ≤24.0 (strength ≤29 kg), (2) BMI 24.1–26.0 (strength ≤30 kg), (3) BMI 26.1–28.0 (strength ≤30 kg), and (4) BMI >28.0 (strength ≤32 kg). For female patients, we classified low if the following was true: (1) BMI ≤23.0 (strength ≤17 kg), (2) BMI 23.1–26.0 (strength ≤17.3 kg), (3) BMI 26.1–29.0 (strength ≤18 kg), and (4) BMI >29.0 (strength ≤21 kg).

### Blood sampling

We have described blood sampling and NMR assessments previously (21). Briefly, approximately 20 ml of blood was collected into vacutainers containing either EDTA or heparin as an anticoagulant. Blood was centrifuged at 3,000 *x* rpm for 10 min at 4°C, and 3–4 ml of plasma was aliquoted and immediately frozen at −80°C. Plasma (600 µl) was analyzed by NMR at Labcorp (Morrisville, NC, USA) as a single batch. NMR spectra were acquired on a Vantera^®^ Clinical Analyzer as previously described (27). The concentration of GlycA, a marker of systemic inflammation (28, 29), was calculated from NMR signal amplitudes of highly mobile protons of *N*-acetylglucosamine residues located on the carbohydrate side chains of circulating acute-phase proteins (e.g., α1-acid glycoprotein, haptoglobin, α1-antitrypsin, α1-antichymotrypsin, and transferrin). Concentrations of lipids, lipoprotein particles, apolipoproteins, and particle sizes were measured using an advanced proprietary deconvolution algorithm (LP4), which provides better subclass resolution than previous algorithms (30–32). Valine, leucine, and isoleucine and their sum (total branched-chain amino acids) were quantified as previously described (33). In addition, glucose, glycine, and alanine were measured using the LP4 algorithm. Trimethylamine N-oxide (TMAO) and betaine were measured by NMR as previously described (34, 35).

### Statistical analysis

We conducted data analyses using SPSS version 28.0 (IBM, Armonk, NY, USA) and the *R* statistics program (R Core Team, 2021). The objective was to determine the association of clinical and biological factors with low physical activity, fitness, and performance characteristics. Descriptive statistics were used to summarize patient characteristics overall and by treatment status. Mean (standard deviation) were reported for continuous characteristics and compared between groups using the Student’s *t*-test. Frequency (proportions) were reported for categorical characteristics and compared between groups using Pearson’s chi-square test. We used logistic regression analyses to determine the association between clinical risk factors and blood measures with the odds of low physical characteristics. Associations were presented as odds ratio (OR), 95% confidence intervals, and the corresponding *p*-values. Univariable models were fit for each clinical risk factor and blood measure. The *p*-values were corrected for multiple testing using the Benjamini–Hochberg procedure to control the false discovery rate at 0.10. Due to a large number of candidate predictors, a least absolute shrinkage and selection operator (LASSO) logistic regression model was also fit to identify a set of informative clinical risk factors and blood measures. LASSO regression performs selection by shrinking (i.e., penalising) the estimated regression coefficient deemed uninformative to precisely zero. The relationship between each selected exposure and an outcome was characterized by a variable importance measure (VIM), which ranks selected exposure based on most to least explanatory power with respect to an outcome. The VIM is calculated as the absolute value of the scaled regression coefficients divided by the largest coefficient in absolute value. Prior to modeling, variables were centered and scaled, and the blood measures were log_2_-transformed because of the right skew in their observed distributions. Blood measures that were highly correlated with other measures were removed from the model to ensure a stable model fit. For all models, the linearity assumption for continuous covariates was assessed using a lack-of-fit test that compared a flexible non-linear fit based on a restricted cubic spline to a linear fit. If evidence of non-linearity was found, an appropriate transformation was used in the model.

## Results

**Demographics**. **Table 1** displays our patient demographics and clinical characteristics. Treatment-naïve and treated patients were of a similar age and had similar body composition at the assessment time. Treated patients had approximately 3.6 years longer time since diagnosis (*p* = 0.001), higher CLL-IPI scores (*p <* 0.001), higher β2-microglobulin levels (*p <* 0.001), and lower white blood cell counts (*p* = 0.005) that were influenced by lower absolute lymphocyte counts (*p* = 0.010). Treated patients had 31.6% more patients classified as RAI stages greater than 0 (*p* = 0.002), and 22.8% more patients with unmutated *IGHV* (*p* = 0.003). All other indices were similar for treatment status.

**Table 1:** Demographics and clinical characteristics of 106 patients assessed in clinic.

	Overall (*N* = 106)	Treatment Naïve (*N* = 47)	Treated (*N* = 59)	*p*-Value
Sex (Male/Female)^1^	67/39	26/21	41/18	0.133
Age (years)	68 (10.2)	67 (9.8)	69 (10.5)	0.287
Race (%)^1^				0.242
African American	6 (5.7%)	1 (2.1%)	5 (8.5%)	
Caucasian	99 (93.4%)	46 (97.9%)	53 (89.8%)	
Other	1 (0.9%)	0 (0%)	1 (1.7%)	
Height (cm)	173.9 (10.8)	172.3 (12.9)	174.8 (8.9)	0.242
Weight (kg)	86.0 (22.9)	81.1 (20.0)	89.2 (24.3)	0.070
BMI (kg/m^2^)	28.2 (5.7)	27.0 (4.8)	28.9 (6.3)	0.090
Clinical Disease Characteristics				
Age at diagnosis (years)	60 (34.9)	61 (9.3)	59.3 (11.4)	0.442
Time since diagnosis (years)	8.3 (5.8)	6.3 (3.3)	9.9 (6.7)	0.001
CLL-IPI Score (a.u.)		1.6 (1.5)	2.9 (2.1)	<0.001
RAI Stage *N* (%)^1^				0.004
0	37 (34.9%)	26 (55.3%)^a^	11 (18.6%)	
I	29 (27.4%)	11 (23.4%)	18 (30.5%)	
II	14 (13.2%)	4 (8.5%)	10 (16.9%)	
III	8 (7.5%)	1 (2.1%)	7 (11.9%)	
IV	14 (13.2%)	4 (8.5%)	10 (16.9%)	
Unknown	4 (3.8%)	1 (2.1%)	3 (5.1%)	
Cytogenetics *N* (%)^1^				
Normal	16 (15.1%)	9 (19.1%)	7 (11.9%)	0.298
Only Del13q	55 (51.9%)	27 (57.4%)	28 (47.5%)	0.307
Any Del11q	20 (18.9%)	5 (10.6%)	15 (25.4%)	0.053
Any Trisomy 12del	16 (15.1%)	6 (12.8%)	10 (16.9%)	0.550
Any Del17p	14 (13.2%)	4 (8.5%)	10 (16.9%)	0.202
del17p and/or TP53mut *N* (%)^1^	14 (13.2%)	5 (10.6%)	9 (15.3%)	0.486
IGHV unmutated *N* (%)^1^	36 (34.0%)	10 (21.3%)	26 (44.1%)	0.003
CD38+ >30% *N* (%)^1^	20 (18.9%)	7 (14.9%)	13 (22.0%)	0.348
Number of Medications	9.3 (4.4)	8.6 (4.1)	9.8 (4.5)	0.157
Clinical Blood Characteristics				
Lactate dehydrogenase (U/L)	199.8 (116.8)	191.7 (112.0)	206.2 (122.1)	0.642
β2-microglobulin (mg/L)	3.1 (1.3)	2.4 (0.7)	3.5 (1.5)	<0.001
Platelets (×10^9^/L)	170.7 (67.4)	177.1 (56.4)	166 (75.2)	0.383
Hemoglobin (g/dl)	13.3 (1.9)	13.2 (1.8)	13.3 (2.0)	0.741
WBC (×10^9^/L)	33.8 (47.8)	48.3 (47.5)	22.2 (45.1)	0.005
Neutrophils	4.1 (1.9)	4.0 (1.4)	4.1 (2.2)	0.742
Lymphocytes	29.3 (47.8)	42.2 (46.2)	18.0 (46.6)	0.010
Monocytes	1.2 (1.6)	1.4 (1.6)	0.9 (1.6)	0.121
Eosinophils	0.2 (0.2)	0.2 (0.2)	0.2 (0.2)	0.879

BMI (body mass index); CLL-IPI [CLL International Prognostic Index in arbitrary unity (a.u.)]; IGHV (immunoglobulin heavy chain variable region). Data are mean (standard deviation) unless otherwise indicated. ^1^Assessed using Chi-square tests. ^a^Significantly different than Treated group.

**Physical characteristics**. Of the physiological and fitness characteristics assessed, treatment-naïve patients had marginally higher (but non-significant) aerobic fitness (*p* = 0.054) and 6MWT distance (*p* = 0.054) and better lower body performance (SPPB: *p* = 0.037) that was characterized by a better ability to repeatedly stand up from sitting (*p* = 0.034) than treated patients (**Table 2**). Upon classification into dysfunctional physical characteristics, the range of dysfunction observed for all patients was between 11% (grip strength) and 49.5% (physical activity levels). Between the groups, 21.8% and 23.7% more treated patients experienced dysfunctional aerobic fitness (eVO_2peak_: *p* = 0.037) and dysfunctional lower body performance scores (SPPB: *p* = 0.012), respectively.

**Table 2:** Physical fitness, function, and activity level characteristics and prevalence of dysfunctions.

	Overall	Treatment Naïve	Treated	*p*-Value	Mean Diff. (95% CI)	Effect Size (*d*)
Aerobic Fitness [eVO_2peak_ (ml/kg/min)]	15.2 (2.2)	15.7 (2.2)	14.8 (2.2)	0.054	0.91 (−0.02, 1.82)	.409
Functional Fitness [6MWT (m)]	444.5 (97.0)	465.7 (94.3)	426.4 (96.5)	0.054	39.3 (−0.62, 79.17)	.412
Lower Body Physical Performance [SPPB Score (max =12)]	10.8 (1.7)	11.2 (1.5)	10.5 (1.8)	0.037	0.73 (0.04, 1.42)	.423
Gait Speed (max = 4)	3.98 (0.15)	3.98 (0.52)	3.98 (0.41)	0.915	−0.01 (−0.06, 0.06)	.000
Chair Stand (max = 4)	3.11 (1.22)	3.40 (1.16)	2.86 (1.23)	0.034	0.54 (0.04, 1.03)	.452
Balance (max = 4)	3.75 (0.58)	3.86 (0.41)	3.66 (0.69)	0.099	0.20 (−0.04, 0.44)	.352
Upper Body Strength [Best Grip Strength (kg)]	36.2 (13.2)	36.4 (13.2)	36.1 (13.4)	0.899	0.35 (−5.14, 5.85)	.002
Physical Activity Levels (SBAS [*N* (%)])^1^						
Inactive	24 (22.6%)	10 (21.3%)	14 (23.7%)	0.577		
Light	25 (23.6%)	11 (44.0%)	14 (23.7%)			
Moderate	29 (27.4%)	15 (31.9%)	14 (23.7%)			
Hard	10 (9.4%)	4 (8.5%)	6 (10.2%)			
Very Hard	11 (10.4%)	6 (12.8%)	5 (8.5%)			
**Prevalence of Dysfunction [ *N* (%)]^1^ **						
Aerobic Fitness (eVO_2peak <_15 ml/kg/min)	41 (45.1%)	14 (33.3%)	27 (55.1%)	0.037		
6MWT - Functional Fitness (Distance ≤400 m)	26 (28.6%)	9 (21.4%)	17 (34.7%)	0.163		
SPPB - Lower Body Physical Performance (Score ≤10)	27 (29.0%)	7 (16.3%)	20 (40.0%)	0.012		
Upper Body Strength	10 (10.9%)	3 (7.1%)	7 (14.0%)	0.293		
Physical Activity Levels (<moderate intensity)	49 (49.5%)	21 (45.7%)	28 (52.8%)	0.476		

6MWT (6-Minute Walk Test); SPPB (Short Physical Performance Battery); SBAS (Stanford Brief Activity Summary). Data are mean (standard deviation) unless otherwise indicated. ^1^Assessed using Chi-square tests. Effect size calculated as Cohen’s d (0.2 = small, 0.5 = medium and 0.8 = large effect size).

**NMR analyses**. Of the 49 analytes measured by NMR, only levels of 10.3-nm HDL particles were different between groups, with treatment-naïve patients having higher levels than treated patients (**Supplementary Table 1**: *p* = 0.043).

**Clinical factors and relations with physical dysfunctions**. **Table 3** describes the relationships between disease duration, treatment status, presence of favorable FISH, age, and BMI and the odds of dysfunctional physical fitness and performance outcomes. Having been diagnosed with CLL for a longer duration was associated with a greater likelihood of having dysfunctional aerobic fitness (eVO_2peak_: OR = 3.56, *p* = 0.002) and dysfunctional lower body physical performance (SPPB: OR = 2.03, *p* = 0.004). Similarly, being treated for CLL was associated with a greater likelihood of having dysfunctional aerobic fitness (eVO_2peak_: OR = 1.57, *p* = 0.036), dysfunctional lower body physical performance (SPPB: OR = 1.85, *p* = 0.011), and dysfunctional upper body strength (Grip Strength: OR = 1.67, *p* = 0.015). Advanced age and BMI were mainly associated with a greater likelihood of dysfunctions in each physical outcome. Although having a favorable FISH profile was not associated with any physical outcomes, having had CLL for less time was associated with less likelihood of having dysfunctional aerobic fitness (eVO_2peak_: OR = 0.66, *p* = 0.002).

**Table 3:** Univariable associations between clinical factors and the odds of having dysfunctional physical characteristics^*^.

	OR (95% CI)	*p*-Value
**Aerobic Fitness (eVO_2peak_)**		
Time Since CLL Diagnosis (years)	Upper: 3.56 (1.37, 9.22) Lower: 0.66 (0.26, 1.71)	0.002
Treatment (yes or no)	1.57 (1.02, 2.40)	0.036
Favorable FISH (yes or no)	1.30 (0.83, 2.03)	0.241
Age (years)	2.76 (1.56, 4.88)	<0.001
BMI (kg/m^2^)	0.86 (0.58, 1.29)	0.470
**Functional Fitness (6MWT)**		
Time Since CLL Diagnosis (years)	1.48 (0.92, 2.39)	0.098
Treatment (yes or no)	1.39 (0.87, 2.23)	0.160
Favorable FISH (yes or no)	1.35 (0.80, 2.28)	0.251
Age (years)	3.96 (1.82, 8.62)	<0.001
BMI (kg/m^2^)	1.24 (0.80, 1.93)	0.336
**Lower Body Physical Performance (SPPB)**		
Time Since CLL Diagnosis (years)	2.03 (1.20, 3.44)	0.004
Treatment (yes or no)	1.85 (1.13, 3.03)	0.011
Favorable FISH (yes or no)	1.47 (0.87, 2.47)	0.132
Age (years)	4.30 (1.98, 9.34)	<0.001
BMI (kg/m^2^)	0.57 (0.34, 0.93)	0.017
**Upper Body Strength (Grip Strength)**		
Time Since CLL Diagnosis (years)	1.26 (0.84, 1.89)	0.261
Treatment (yes or no)	1.67 (1.10, 2.55)	0.015
Favorable FISH (yes or no)	1.23 (0.80, 1.89)	0.344
Age (years)	Upper: 0.53 (0.21, 1.33) Lower: 7.41 (2.50, 22.01)	<0.001
BMI (kg/m^2^)	2.98 (1.70, 5.24)	<0.001
**Physical Activity Levels (SBAS)**		
Time Since CLL Diagnosis (years)	0.87 (0.59, 1.29)	0.493
Treatment (yes or no)	1.15 (0.78, 1.71)	0.476
Favorable FISH (yes or no)	0.95 (0.63, 1.44)	0.823
Age (years)	1.01 (0.68, 1.50)	0.950
BMI (kg/m^2^)	1.68 (1.09, 2.59)	0.014

FISH (fluorescent in situ hybridization); BMI (body mass index); OR (odds ratio); CI (confidence interval). *All continuous covariates were log2-transformed, centred, and scaled prior to modeling.

**Circulating factors and relations with physical dysfunctions**. We next determined the relationships between physical outcomes and circulating blood parameters (**Table 4**). Eight factors were associated with aerobic capacity following FDR adjustment. Specifically, higher concentrations of citrate (OR = 2.01, FDR *p* = 0.03) and larger average HDL particle size (OR = 1.79, FDR *p* = 0.069) were associated with a greater likelihood of having dysfunctional aerobic fitness. Contrastingly, higher total protein (OR = 0.51, FDR *p* = 0.069), higher hemoglobin (OR = 0.50, *p* = 0.03), higher H2P HDL subspecies (OR = 0.39, FDR *p* = 0.028), higher total HDL particle concentration (OR = 0.34, FDR *p* = 0.0011), higher total small HDL particle concentrations (OR = 0.32, FDR *p* = 0.004), and higher concentrations of HDL particles <9 nm in size (OR = 0.24, FDR *p <* 0.001) were all associated with less likelihood of having dysfunctional aerobic fitness. Of the remaining relationships, we observed that only higher levels of TMAO (OR = 2.31, FDR *p* = 0.064) were associated with a greater likelihood of having dysfunctional grip strength.

**Table 4:** Univariable associations between circulating blood measures and the odds of having dysfunctional physical characteristics^*^.

	OR (95% CI)	Unadjusted *p*-Value	FDR *p*-Value
**Aerobic Fitness (eVO_2peak_)**			
Citrate (μmol/L)	2.01 (1.22, 3.31)	0.003	0.030
Average HDL-P size (nm)	1.79 (1.13, 2.83)	0.009	0.069
Total Protein (a.u.)	0.51 (0.29, 0.88)	0.010	0.069
Hemoglobin (g/dl)	0.50 (0.31, 0.82)	0.003	0.030
7.8 nm HDL Subspecies H2P (μmol/L)	0.39 (0.20, 0.75)	0.002	0.028
Total HDL-P (nmol/L)	0.34 (0.17, 0.67)	0.001	0.001
Total small HDL-P (nmol/L)	0.32 (0.16, 0.61)	<0.001	0.004
Total HDL-P <9 nm (μmol/L)	0.24 (0.12, 0.49)	<0.001	<0.001
**Functional Fitness (6MWT)**			
GlycA (μmol/L)	1.98 (1.13, 3.46)	0.012	0.156
Citrate (μmol/L)	1.88 (1.09, 3.21)	0.015	0.156
Neutrophil counts (×10^9^/L)	1.76 (1.02, 3.03)	0.030	0.209
Total Protein (a.u.)	0.55 (0.31, 0.95)	0.027	0.209
7.8 nm HDL Subspecies H2P (μmol/L)	0.48 (0.25, 0.90)	0.016	0.156
Total small HDL-P (nmol/L)	0.41 (0.22, 0.78)	0.004	0.114
Total HDL-P <9 nm (μmol/L)	0.40 (0.21, 0.77)	0.003	0.114
**Lower Body Physical Performance (SPPB)**			
Total medium LDL particles (nmol/L)	Upper: 2.21 (1.10, 4.45) Lower: 0.00 (0.00, 0.34)	0.030	0.307
Average HDL-P size (nm)	1.64 (1.04, 2.59)	0.031	0.307
Hemoglobin (g/dl)	0.61 (0.38, 0.99)	0.041	0.307
Large TRLP (nmol/L)	0.60 (0.37, 0.98)	0.038	0.307
Total small HDL-P (nmol/L)	0.46 (0.25, 0.84)	0.008	0.158
Total HDL-P <9 nm (μmol/L)	0.43 (0.23, 0.80)	0.005	0.153
**Upper Body Strength (Grip Strength)**			
TMAO (μM)	2.31 (1.31, 4.07)	0.001	0.064
Average LDL-P size (nm)	Upper: 1.93 (0.64, 5.88) Lower: 0.40 (0.18, 0.88)	0.034	0.233
10.3 nm HDL Subspecies H5P (μmol/L)	0.60 (0.37, 0.97)	0.022	0.233
HDL-C (mg/dl)	0.59 (0.35, 1.00)	0.042	0.233
Total large LDL-P (nmol/L)	0.59 (0.36, 0.97)	0.029	0.233
7.4 nm HDL Subspecies H1P (μmol/L)	0.58 (0.34, 0.98)	0.027	0.233
Lymphocyte counts (×10^9^/L)	0.57 (0.35, 0.93)	0.016	0.233
Total HDL-P (nmol/L)	0.56 (0.32, 1.00)	0.043	0.233
**Physical Activity Levels (SBAS)**			
Very small TRLP (nmol/L)	Upper: 0.21 (0.04, 1.05) Lower: 3.70 (0.82, 16.69)	0.018	0.513

HDL-P (high-density lipoprotein particles); LDL-P (low-density lipoprotein particles); HDL-C (high-density lipoprotein cholesterol); TRLP (triglyceride-rich lipoprotein). OR (odds ratio); CI (confidence interval). *All continuous covariates were log2-transformed, centred, and scaled prior to modeling; 49 tests were performed for each physical characteristic, and only tests with unadjusted p-value < 0.05 were reported here.

**Multivariable relationships**. In **Table 5**, we performed a LASSO-penalized logistic regression that included all the biological and clinical factors to identify informative markers for the odds of having each of the physical dysfunctions. The VIM ranks selected exposure based on most to least explanatory power with respect to an outcome. Here, we discuss data with a VIM greater than 0.200. For aerobic fitness, we identified five factors with a VIM greater than 0.200. For the odds of having lower aerobic fitness, circulating levels of the 7.8-nm HDL subspecies H2P were the most informative (VIM = 1.000). Relative to H2P levels, age (VIM = 0.481) and hemoglobin levels (VIM = 0.437) were similarly informative about the odds of having low aerobic fitness, and both contributed more information than favorable FISH (VIM = 0.234) and lymphocyte counts (VIM = 0.206). For functional fitness (6MWT), we identified three factors with a VIM > 0.200. For the odds of having low functional fitness, circulating levels of total HDL particles (VIM = 1.000) were the most informative. Relative to HDL particle levels, age (VIM = 0.901) contributed the most additional information and contributed more information than hemoglobin levels (VIM = 0.291) for the odds of low functional fitness. For lower body physical performance (SPPB), we identified seven factors with a VIM > 0.200. For the odds of having worse lower body performance scores, age (VIM = 0.1000) was the most informative. Relative to age, BMI (VIM =0.683) and disease duration (VIM = 0.680) contributed similar additional amounts of information for the odds of low SPPB scores. Treatment status (VIM = 0.490) and levels of the 10.3-nm HDL subspecies H5P (VIM = 0.401) contributed additional information, and more information than FISH status (0.219) and levels of very large triglyceride-rich lipoproteins (VIM = 0.212). For grip strength, we identified eight factors with a VIM >0.200. BMI (VIM = 1.000) was the most informative for the odds of having low grip strength. Relative to BMI, age (VIM = 0.790) contributed additional information, and more than higher levels of citrate (VIM = 0.508). Levels of the 10.3-nm HDL subspecies (VIM = 0.383), average LDL particle size (VIM = 0.367), lower levels of citrate (VIM = 0.251), levels of TMAO (VIM = 0.259), and absolute leukocyte counts (VIM = 0.240) all contributed less information than higher levels of citrate and age for the odds of low grip strength. The LASSO-penalized logistic regression revealed no additionally informative factors for the odds of having low physical activity levels.

**Table 5:** Variable selection results of clinical and circulating factors for the odds of having dysfunctional physical characteristics.

	Cut point	VIM
**Aerobic Fitness (eVO_2peak_)**		
7.8 nm HDL-P Subspecies H2P (μmol/L)		1.000
Age (years)		0.481
Hemoglobin (g/dl)		0.437
Favorable FISH (yes or no)		0.234
Lymphocyte count (×10^9^/L)		0.206
9.5 nm HDL-P Subspecies H4P (μmol/L)		0.113
Glycine (μmol/L)	Lower	0.103
Medium LDL-P [20.5–21.4nm (nmol/L)]	Upper	0.056
**Functional Fitness (6MWT)**		
Total HDL particles (μmol/L)		1.000
Age (years)		0.901
Hemoglobin (g/dl)		0.291
**Lower Body Physical Performance (SPPB)**		
Age (years)		1.000
BMI (kg/m^2^)		0.683
Time Since CLL Diagnosis (years)		0.680
Treatment (yes or no)		0.490
10.3 nm HDL-P Subspecies H5P (μmol/L)		0.401
Favorable FISH (yes or no)		0.219
Very large TRLP [90–240 nm (nmol/L)]		0.212
7.8 nm HDL-P Subspecies H2P (μmol/L)		0.135
Leukocyte count (×10^9^/L)		0.043
**Upper Body Strength (Grip Strength)**		
BMI (kg/m^2^)		1.000
Age (years)		0.790
Citrate (μmol/L)	Upper	0.508
10.3nm HDL-P Subspecies H5P (μmol/L)		0.383
Average LDL particle size (19–22.5 nm)	Lower	0.367
Citrate (μmol/L)	Lower	0.251
TMAO (μM)		0.249
Leukocyte count (×10^9^/L)		0.240
Lymphocyte count (×10^9^/L)		0.186
Valine (μmol/L)		0.122
TRLP (nmol/L)		0.106
Creatinine (mg/dl)		0.076
Large LDL-P (μmol/L)		0.051
Hemoglobin (g/dl)		0.041
8.7 nm HDL-P Subspecies H3P (μmol/L)		0.032
**Physical Activity Levels (SBAS)^a^**		

VIM (variable importance measure); HDL (high-density lipoprotein); FISH (fluorescent in situ hybridization); BMI (body mass index); TRLP (triglyceride-rich lipoprotein); LDL (low-density lipoprotein); TMAO (trimethylamine N-oxide). ^a^No biomarkers or clinical factors were associated with SBAS.

## Discussion

CLL is associated with a progressive worsening of major bodily systems that increases mortality risk from infections, hospitalizations, and secondary malignancies. A clinically significant factor that worsens these risks is low physiological fitness that predisposes patients to higher risks of frailty. For the first time that we are aware of, we have assessed clinical and circulating blood factors and how they relate to physical fitness, physical activity levels, and physical performance characteristics in a heterogeneous population of CLL patients. We show two main clinical and biological factors that are associated with dysfunctional fitness—CLL treatment and components of lipids and lipoproteins. We observed expected clinical differences between patients who had or had not received CLL specific treatments. We also observed more treated patients with dysfunctional SPPB status and aerobic fitness. In univariate regression analyses, we found that time since CLL diagnosis and treatment were associated with the likelihood of having several dysfunctional physical characteristics. We also found that several indicators of differential lipid metabolism, including HDL components and citrate, were associated with the probability of having dysfunctional aerobic physical fitness. Following multivariable analyses, HDL components were more informative than the clinical indices considered in our analyses for the odds of dysfunctional aerobic fitness and 6-min walk distance. Taken together, we present evidence of the relationships between clinical factors, biological factors, and physical fitness characteristics that could potentially prompt early involvement of ancillary support services, including dieticians, physical therapists, and exercise physiologists.

For the first time that we are aware of, we show that lipids and lipoproteins are associated with the likelihood of dysfunctional fitness in CLL. We demonstrate that higher levels of small HDL lipoprotein components are associated with a lower likelihood of having dysfunctional fitness levels, suggesting that HDL may play an important role in the fitness of our CLL patients. Known factors that typically increase muscle uptake of lipids, alter systemic and cellular lipid metabolism, and promote higher aerobic fitness and HDL levels include a healthy diet, increased physical activity, and exercise training (36, 37). Both aerobic and resistance exercise training induce muscle lipoprotein lipase (LPL) mRNA, protein mass, and enzyme activity (36, 38, 39). Muscle LPL hydrolyzes triglycerides from circulating chylomicrons and very-low-density lipoproteins (VLDL), releasing free fatty acids (FFA) and esterified cholesterol. The FFA, including those released from adipocytes during exercise, then enter muscle cells for energy production and storage, while esterified cholesterol is taken up by HDL particles and cleared from the system. Whether this occurs in CLL patients and what effect this would have on CLL cells is not known.

Dyslipidemia and hypercholesterolemia have complex and not fully understood roles in the pathogenesis of CLL (40, 41). HDL functions to remove esterified cholesterol following lipolysis of chylomicron and VLDL triglycerides (42). In the absence of sufficient HDL levels, cholesterol will be utilized by CLL cells and contributes to the increased risk of cardiovascular disease observed in adults with CLL (43, 44). As such, CLL cells have a preferential energy utilization of fatty acids and cholesterol, delivered primarily by chylomicrons, VLDL, and LDL, respectively (20, 44). Thus, CLL cells can reduce the amount of these necessary energy substrates delivered to healthy tissues and organs (20). Corroborating this, we observed that higher levels of circulating citrate is associated with a greater likelihood of dysfunctional aerobic fitness. Citrate is a crucial regulator of several functions that benefit CLL cells, including fatty acid synthesis/metabolism and biosynthetic processes such as proliferation (45, 46). It is unclear whether the extracellular citrate is transported into CLL cells for fatty acid synthesis (39), or whether it is expelled from CLL cells because lipoprotein uptake *via* LPL has reached peak saturation (20). CLL cells overexpress both the LDL-receptor and LPL in the cytosol and cell membrane, facilitating lipoprotein uptake into lipid vacuoles for fatty acid synthesis (20, 44). In high-risk patients such as unmutated *IGHV*, LPL expression may be less than that in low-risk patients but with higher enzymatic activity (47). Although the mechanisms remain largely unknown, the prognostic value of LPL expression and the benefits of cholesterol inhibitors such as statins and exercise training are becoming more apparent (13, 44, 48, 49). The full implications of our observations are not known, but it is possible that CLL cells are misappropriating metabolic fuels that promote their survival, likely to the detriment of healthy tissues such as skeletal muscle that require lipid fuels (20).

In solid tumors, dysregulated lipid metabolism contributes to cancer cachexia and the depletion of energy stores available to maintain muscle health (50). One potential mechanism of cancer cachexia is the hyperactivation and expression of LPL, which, if pharmaceutically reduced, modulates metabolic dysregulation, leading to solid tumor cachexia (51). Since CLL is a chronic, often slow-progressing cancer, it is plausible that some of the same mechanisms underpinning the rapid development of solid tumor cancer cachexia are present in CLL and contribute to low physical fitness. Further studies are required to confirm this. However, we recently observed evidence that would suggest that exercise may be redirecting lipids from CLL cells to skeletal muscle (52). Following 12 weeks of aerobic plus resistance exercise training, we observed significant increases in CLL patients’ muscle strength, and these strength changes were associated with lower numbers of circulating CLL cells (52). We also observed that treatment-naïve CLL patients with higher physical fitness levels have circulating blood factors that reduce the growth of a CLL-like cell line (21). Specifically, higher fitness was associated with higher HDL levels and lower triglyceride levels. We postulate that these observations are similar to those made in the present study and highlight the importance of HDL in modulating lipid metabolism in CLL. Further studies are required to determine whether exercise and higher fitness lipoprotein differences directly affect CLL cell function (for example, reducing tumor growth and redirecting energy substrates away from CLL cells to muscle cells).

The strengths of our study include a large number of treated and treatment-naïve patients and the comprehensive characterization of plasma biomarkers. Additionally, a major strength of this study was the ability to efficiently test these patients in the clinic during real-world situations. Limitations of our study include the following: (1) the study is observational; (2) we have not explored any of the potential mechanisms discussed, including the levels of LPL or lipoprotein receptors in CLL cell samples; and (3) while we conducted a comprehensive NMR-based lipoprotein characterization, we have not determined the entire circulating metabolome, including different fatty acid levels, to provide additional information about energy utilization. Additionally, we were unable to determine the relationships between physical dysfunction, NMR measures, and supplement use or comorbidities. Given both supplement use and comorbidities are high in people with CLL, especially for those undergoing treatment, there is the possibility that these factors will influence our observations (53, 54). Finally, we did not determine associations between outcomes and treatment factors, including the type of treatment (e.g., BTKi versus BCL2i) or the time on treatment dynamics. Although it is likely that these, too, could affect our observations, we did not have a sufficient sample size to power such sub-studies. There are two crucial future directions for our research: (*i*) explorations of the likely mechanisms associated with metabolic dysfunction and physical fitness in CLL, and (*ii*) attempts to experimentally increase HDL levels and determine if health improvements occur.

## Conclusion

In conclusion, we provide evidence that CLL clinical factors (i.e., treatment status and disease duration) and several lipid and lipoprotein levels (i.e., HDL components) are associated with physical performance and fitness levels. Our data suggest that prior CLL treatment may alter levels of HDL components and that low levels of small HDL components are associated with dysfunctional physical fitness. Those with higher levels of small HDL components have less likelihood of low physical fitness, suggesting that interventions that increase HDL levels may benefit adults with CLL. Coupled with our recent CLL exercise intervention that increased muscle strength and slowed leukemia cell increases, we postulate that exercise is one such intervention. Future studies should aim to understand the role of modifying energy balance in CLL patients and whether altering the energy balance of leukemia cells and healthy tissues may be a clinically meaningful adjuvant therapy.

## Data availability statement

The raw data supporting the conclusions of this article will be made available by the authors, without undue reservation.

## Ethics statement

This study was reviewed and approved by Duke University Medical Center Institutional Review Board. The patients/participants provided their written informed consent to participate in this study.

## Author contributions

DBB, DB, JW, and AS conceived and designed the study and experimental approach. EG and MG completed the NMR spectroscopy and data analysis. MD, DT, and TS identified and completed physical testing on the patients. BN and MN conducted the statistical analyses. GM managed the database helped with data interpretation. EH consulted on the physical assessment results and data interpretation. AA analyzed and wrote the demographic data interpretation. All authors contributed to the article and approved the submitted version.

## Funding

This work was supported by a Duke Claude D. Pepper Older Americans Independence Center Pilot Study Award (National Institutes of Health, National Institute on Aging P30-AG028716), National Institutes of Health, National Heart, Lung, and Blood Institute T32 grant (T32HL007057), the Durham Veterans Affairs Medical Center Research Service, and an American Society of Hematology Scholars Award.

## Acknowledgments

The research team acknowledges the support of the Duke Cancer Center and the support provided by nurses and clinical staff during the execution of the study. Finally, we appreciate the participants who kindly gave up their time to participate in this study.

## Conflict of interest

EG and MC are employees of Labcorp.

DB has been a consultant, scientific advisory board member, and site clinical trial Principal Investigator (PI) (grant paid to institution) for AbbVie, Genentech, and Verastem; scientific advisory board member and site clinical trial PI (grant paid to institution) for ArQule and TG Therapeutics; site clinical trial PI (grant paid to institution) for Ascentage, BeiGene, DTRM, Juno/Celgene/BMS, MEI Pharma, and Tolero; consultant and site clinical trial PI (grant paid to institution) for AstraZeneca and Pharmacyclics; consultant and scientific advisory board member for Pfizer; consultant for Teva; National Comprehensive Cancer Network panel member; and has participated in the informCLL registry steering committee (AbbVie), REAL registry steering committee (Verastem), and Biosimilars outcomes research panel (Pfizer).

The remaining authors declare that the research was conducted in the absence of any commercial or financial relationships that could be construed as a potential conflict of interest.

## Publisher’s note

All claims expressed in this article are solely those of the authors and do not necessarily represent those of their affiliated organizations, or those of the publisher, the editors and the reviewers. Any product that may be evaluated in this article, or claim that may be made by its manufacturer, is not guaranteed or endorsed by the publisher.
