# Supplementary material for: Associations of clinical and circulating metabolic biomarkers with low physical fitness and function in adults with chronic lymphocytic leukemia
Source: Front Oncol. 2022 Aug 3;12:933619. doi: 10.3389/fonc.2022.933619 (PMC9381973; doi:10.3389/fonc.2022.933619)
Supplement: Supplementary file 1 [file Table_1.docx]

Supplementary Table 1. Total and group differences for all NMR Measures^1^

|  | Overall  (N=106) | Treatment  Naïve (N=47) | Treated  (N=59) | *p*-Value |
| --- | --- | --- | --- | --- |
| **Inflammation** |  |  |  |  |
| GlycA (µmol/L) | 421.2 (370.1 – 456.4) | 418.0 (355.5 – 440.8) | 424.6 (384.8 – 463.2) | 0.185 |
| Betaine (µM) | 47.4 (39.3 – 60.2) | 52.9 (36.0 – 64.3) | 45.1 (39.7 – 56.8) | 0.314 |
| TMAO (µM) | 3.7 (2.0 – 6.6) | 3.8 (1.8 – 9.1) | 3.6 (2.0 – 5.8) | 0.433 |
| **Metabolic Intermediaries** |  |  |  |  |
| Glucose (mg/dL) | 80.0 (70.0 – 94.0) | 84.0 (70.0 – 99.0) | 79.0 (71.0 – 94.0) | 0.767 |
| Citrate (µmol/L) | 141.0 (119.8 – 164.3) | 139.0 (126.0 – 168.0) | 142.0 (114.0 – 162.0) | 0.604 |
| **Triglycerides** |  |  |  |  |
| Total Triglycerides (mg/dL) | 124.5 (81.3 – 166.5) | 124.0 (75.0 – 181.0) | 125.0 (84.0 – 163.0) | 0.942 |
| TRL Triglycerides (mg/dL) | 102.5 (62.5 – 145.0) | 104.0 (63.0 – 156.0) | 95.0 (61.0 – 140.0) | 0.939 |
| Total TRLP (nmol/L) | 141.0 (98.8 – 194.4) | 147.3 (86.3 – 212.2) | 130.6 (102.6 – 185.1) | 1.000 |
| Very Large (nmol/L) | 0.3 (0.1 – 0.5) | 0.3 (0.1 – 0.5) | 0.3 (0.1 – 0.5) | 0.748 |
| Large (nmol/L) | 3.0 (0.5 – 7.7) | 4.2 (0.6 – 7.8) | 1.6 (0.2 – 6.9) | 0.115 |
| Medium (nmol/L) | 16.5 (6.6 – 29.0) | 15.3 (5.1 – 31.2) | 17.7 (8.7 – 27.7) | 0.498 |
| Small (nmol/L) | 33.8 (19.7 – 62.2) | 33.3 (19.5 – 70.0) | 34.3 (19.7 – 60.0) | 0.686 |
| Very Small (nmol/L) | 70.9 (37.0 – 111.4) | 68.8 (34.0 – 105.4) | 71.4 (44.7 – 112.8) | 0.717 |
| **Cholesterol (mg/dL)** |  |  |  |  |
| Total Cholesterol | 183.0 (154.8 – 217.0) | 185.0 (147.0 – 217.0) | 179.0 (157.0 – 216.0) | 0.944 |
| TRL Cholesterol | 26.5 (19.0 – 35.3) | 27.0 (14.0 – 38.0) | 26.0 (20.0 – 32.0) | 0.947 |
| LDL Cholesterol | 98.0 (79.8 – 126.3) | 100.0 (78.0 – 125.0) | 96.0 (86.0 – 127.0) | 0.770 |
| HDL Cholesterol | 53.0 (43.8 – 62.0) | 55.0 (43.0 – 63.0) | 51.0 (44.0 – 59.0) | 0.277 |
| HDL-P (µmol/L) |  |  |  |  |
| Total | 22.7 (19.8 – 25.1) | 22.8 (19.9 – 25.8) | 22.3 (19.6 – 24.5) | 0.334 |
| Large | 1.7 (1.3 – 2.7) | 1.6 (1.3 – 2.9) | 1.7 (1.2 – 2.3) | 0.378 |
| Medium | 4.7 (3.5 – 5.7) | 4.0 (3.0 – 5.5) | 4.7 (3.8 – 5.9) | 0.251 |
| Small | 15.9 (13.0 – 18.3) | 16.4 (12.7 – 18.9) | 15.6 (13.2 – 17.4) | 0.294 |
| Small Particles <9nm | 18.6 (15.8 – 21.0) | 18.6 (16.4 – 21.2) | 18.6 (15.7 – 21.0) | 0.677 |
| H7P Subspecies | 0.2 (0.1 – 0.4) | 0.2 (0.1 – 0.5) | 0.2 (0.1 – 0.4) | 0.340 |
| H6P Subspecies | 0.6 (0.2 – 1.1) | 0.4 (0.1 – 1.2) | 0.7 (0.3 – 1.1) | 0.043 |
| H5P Subspecies | 0.8 (0.4 – 1.3) | 1.1 (0.5 – 1.5) | 0.7 (0.4 – 2.2) | 0.490 |
| H4P Subspecies | 1.5 (0.9 – 2.1) | 1.3 (0.9 – 2.0) | 1.6 (1.0 – 2.2) | 0.370 |
| H3P Subspecies | 3.1 (1.9 – 4.0) | 2.9 (1.6 – 3.7) | 3.2 (1.9 – 4.2) | 0.604 |
| H2P Subspecies | 11.9 (9.5 – 14.3) | 12.5 (9.5 – 14.5) | 11.7 (9.8 – 14.1) | 0.121 |
| H1P Subspecies | 3.3 (1.9 – 4.9) | 3.5 (2.6 – 5.6) | 2.7 (1.7 – 4.8) | 0.421 |
| Apolipoprotein A1 (mg/dL) | 140.0 (120.0 – 158.8) | 147.0 (120.0 – 166.0) | 138.0 (120.0 – 155.0) | 0.329 |
| LDL-P (nmol/L) |  |  |  |  |
| Total | 1445.0 (1207.0 – 1828.5) | 1449.0 (1130.0 – 1825.0) | 1432.0 (1245.0 – 1839.0) | 0.654 |
| Large | 127.5 (57.5 – 259.3) | 153.0 (59.0 – 345.0) | 116.0 (56.0 – 230.0) | 0.321 |
| Medium | 4.5 (0.0 – 201.8) | 3.0 (0.0 – 235.0) | 6.0 (0.0 – 192.0) | 0.599 |
| Small | 1168.0 (869.0 – 1422.5) | 1152.0 (821.0 – 1400.0) | 1170.0 (907.0 – 1430.0) | 0.234 |
| Apolipoprotein B (mg/dL) | 84.0 (69.0 – 103.3) | 84.0 (66.0 – 103.0) | 84.0 (69.0 – 104.0) | 0.736 |
| **Lipoprotein Size (nm)** |  |  |  |  |
| TRL | 46.1 (41.3 – 50.6) | 47.3 (42.1 – 50.8) | 45.0 (40.5 – 50.0) | 0.421 |
| LDL | 20.5 (20.1 – 20.8) | 20.6 (19.9 – 20.8) | 20.5 (20.1 – 20.7) | 0.606 |
| HDL | 8.9 (8.6 – 9.2) | 8.9 (8.6 – 9.3) | 8.8 (8.6 – 9.1) | 0.878 |
| **Amino Acids (µmol/L)** |  |  |  |  |
| Total BCAA | 373.0 (311.0 – 449.3) | 366.0 (307.0 – 463.0) | 383.0 (311.0 – 449.0) | 0.329 |
| Valine | 206.0 (171.8 – 244.3) | 204.0 (171.0 – 243.0) | 209.0 (172.0 – 245.0) | 0.995 |
| Leucine | 124.0 (89.8 – 150.3) | 129.0 (91.0 – 154.0) | 119.0 (89.0 – 147.0) | 0.624 |
| Isoleucine | 44.5 (29.0 – 57.3) | 45.0 (25.0 – 56.0) | 44.0 (31.0 – 59.0) | 0.613 |
| Glycine | 215.4 (174.9 – 280.6) | 209.1 (173.3 – 268.3) | 232.7 (179.6 – 291.1) | 0.929 |
| Alanine | 381.5 (338.5 – 452.0) | 384.0 (346.0 – 459.0) | 376.0 (314.0 – 443.0) | 0.368 |

TMAO (Trimethylamine N-oxide); TRL (Triglyceride Rich Lipoprotein); TRLP (Triglyceride Rich Lipoprotein Particles); LDL (Low-Density Lipoprotein); HDL (High-Density Lipoprotein); HDL-P (High-Density Lipoprotein Particles); LDL-P (Low-Density Lipoprotein Particles); BCAA (Branch Chained Amino Acids). ^1^Data were analyzed by Mann-Whitney U Test and data are median (25^th^ – 75^th^ Quartile)
